# Aquaporin-4 Expression Switches from White to Gray Matter Regions during Postnatal Development of the Central Nervous System

**DOI:** 10.3390/ijms24033048

**Published:** 2023-02-03

**Authors:** Francisco Mayo, Lourdes González-Vinceiro, Laura Hiraldo-González, Claudia Calle-Castillejo, Sara Morales-Alvarez, Reposo Ramírez-Lorca, Miriam Echevarría

**Affiliations:** 1 Institute of Biomedicine of Seville (IBiS), Virgen del Rocío University Hospital, (HUVR)/Spanish National Research Council (CSIC)/University of Seville, 41013 Seville, Spain; 2 Department of Physiology and Biophysics, University of Seville, 41009 Seville, Spain

**Keywords:** aquaporins, AQP4, central nervous system (CNS), gray matter, white matter, oligodendrocytes, astrocytes, oligodendrogenesis, myelination

## Abstract

Aquaporin-4 (AQP4) is the most abundant water channel in the central nervous system and plays a fundamental role in maintaining water homeostasis there. In adult mice, AQP4 is located mainly in ependymal cells, in the endfeet of perivascular astrocytes, and in the glia limitans. Meanwhile, its expression, location, and function throughout postnatal development remain largely unknown. Here, the expression of AQP4 mRNA was studied by in situ hybridization and RT-qPCR, and the localization and amount of protein was studied by immunofluorescence and western blotting, both in the brain and spinal cord. For this, wild-type mice of the C57BL/6 line, aged 1, 3, 7, 11, 20, and 60 days, and 18 months were used. The results showed a change in both the expression and location of AQP4 in postnatal development compared to those during adult life. In the early stages of postnatal development it appears in highly myelinated areas, such as the corpus callosum or cerebellum, and as the animal grows, it disappears from these areas, passing through the cortical regions of the forebrain and concentrating around the blood vessels. These findings suggest an unprecedented possible role for AQP4 in the early cell differentiation process, during the first days of life in the newborn animal, which will lead to myelination.

## 1. Introduction

In the brain, AQP4 is expressed in the basolateral membranes of ependymal cells that line the ventricles, in the foot processes of perivascular astrocytes that help to form the blood brain barrier (BBB), in subpial astrocytes of the glia limitans and subependymal glia, and in vascular endothelial cells, but not in neurons [1,2,3,4]. It has been found in other structures of the brain such as circumventricular organs, the magnocellular hypothalamic nucleus, the dentate gyrus, or the temporal neocortex [5], in addition to being expressed in the spinal cord [6,7], an important zone also included in the CNS. Astrocytes do not have tight junctions in their plasma membrane that could prevent the lateral diffusion of structural molecules inside the membrane. Thus, to allow the polarized and specific localization of proteins in their cell plasma membrane, as described for AQP4, the presence of a plasma membrane anchoring mechanism is necessary.

In the adult brain of mice, AQP4 is anchored to the cell membrane in the endfeet of astrocytes by coexpression with dystroglycan, and binds intracellularly with alpha-syntrophin, alpha-dystrophin, and dystrobrevin, and toward the extracellular side with laminin and agrin [2,8,9], forming part of a protein scaffold collectively called the dystrophin-associated protein complex (DAPC) [8,10]. A more detailed description: AQP4 presents at its C-terminal end the SSV sequence (serine-serine-valine), which is capable of binding to the PDZ domain of syntrophins (syn), a cytoplasmic membrane protein [11]. Syntrophins can bind to dystrobrevin, which in turn binds to delta-dystrophin, which is part of a large membrane complex that connects the cytoskeleton to the extracellular matrix (ECM). Specifically, dystrophin forms bridges between actin filaments and the beta-dystroglycan transmembrane protein. This complex is especially relevant in the CNS, as it determines the polarization of AQP4 expression in perivascular astrocytes that form the BBB [8,12].

In contrast to this detailed knowledge of the expression of AQP4 in the adult brain, there is little information about its postnatal expression, and the role of AQP4 in embryonic and postnatal development [13,14]. Several hypotheses have tried to respond to the possible functions of AQP4 during these phases. A plausible one is that AQP4 appears to be involved in the decrease in the brain water content observed during this initial period after birth. This idea would be supported by the delay observed in the reduction in the brain water content in AQP4-KO [15], but also in the glial conditional AQP4-KO model [16]. Analysis of the postnatal development of the molecular complex that allows the polarization of astrocytes, revealed that the signal for AQP4 was detected on postnatal day 7 (P7), in the subpial zone, and also around the vessels. Later, between P13 and P21, the labeling in the neuropil decreased, further highlighting the clear appearance of AQP4 in the two areas indicated. The general understanding we have today is that the presence of AQP4 is associated with the appearance of astrocytes in the brain, which appear to contribute to postnatal angiogenesis and BBB formation [17]. To support this, previous studies have also shown that the highly polarized expression of AQP4 seen in adulthood around the perivascular glia is completed at P14, paralleling the BBB formation in mice [15].

Astrocytes are the most abundant cell type in the CNS and have a wide variety of functions, such as maintaining the impermeability of the BBB, the formation of neuronal circuits, and playing a key role in brain metabolic regulation, among others [18]. Despite this, there are still many doubts about the characterization of their development and the molecular processes that support it. At birth, due to brain growth during the postnatal period, the number of glial cells increases by six to eight times during the first three weeks of life. Radial glia cells are those that originally function as stem cells, that give rise to all types of CNS cells, including astrocytes and ependymal cells. These essentially make up the membranes of the CNS/CSF interface, which express AQP4 especially abundantly. In the postnatal stage, differentiation occurs between protoplasmic astrocytes (PA) of gray matter (GM), and fibrous astrocytes (FA) of white matter (WM). As astrocytes mature, their transcriptional profiles change drastically, so they express combinations of molecules characteristic of each stage [18]. In terms of this work, in the developing brain of mice, AQP4 expression has been observed at the earliest on embryonic day 16 (E16) [14]. However, only after birth does AQP4 expression begin to be observed in astrocytes, and perivascular polarized expression does not appear until the first two weeks of the postnatal stage. More studies are needed to better understand how AQP4 expression levels vary from postnatal development to adulthood.

In the present work, we have analyzed the expression and redistribution of AQP4 throughout postnatal development, with a particular focus from P7 to adult life. The expression of AQP4 mRNA was studied by in situ hybridization and RT-qPCR, and the localization of the protein was studied by immunofluorescence analysis and western blotting, both in the brain and in the spinal cord of animals aged 1, 3, 7, 11, 20, and 60 days, and 18 months.

## 2. Results

### 2.1. Expression of AQP4 mRNA by In Situ Hybridization (ISH) and RT-qPCR

**Figure 1:**
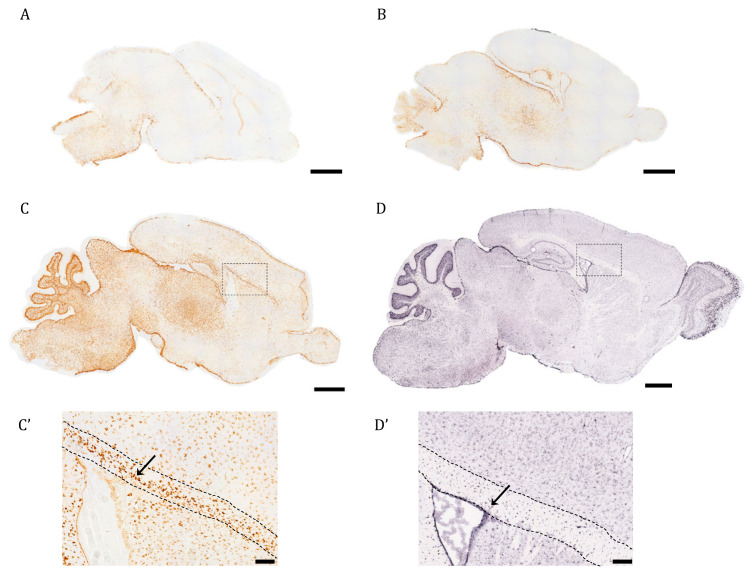
The pattern of AQP4 mRNA induction in brain during the first postnatal week differs from the adult transcript distribution. Identification of AQP4 mRNA molecules by in situ hybridization in cerebral sagittal sections from neonatal mice at P1 (**A**), P3 (**B**), P7 (**C**), and juvenile mice at P56 ((**D**), image obtained from Allen Brain Atlas). High magnification of corpus callosum (comprised between the dashed point lines), surrounding areas of P7 and P56 brains are shown in (**C’**) and (**D’**). The arrows indicate AQP4+ stained cells in both (**C’**,**D’**). Scale bar = 1 mm (**A**–**D**) and 150 µm in the insets (**C’**,**D’**).

In this work, we were interested in profiling the spatiotemporal distribution of AQP4 during postnatal development and adult life in the full mouse brain. As a first approximation, the ISH technique was used in brain sagittal sections of early postnatal mice to evaluate when and where AQP4 mRNA expression begins within the different cerebral regions (Figure 1). On postnatal day 1 (P1), labeling is scarce, except for the area of the brainstem (BS), where the signal begins to be detected mainly in the area closest to the edges (Figure 1A). In fact, on embryonic day 16, the incipient signal in the BS can also be observed (see Supplementary Figure S1). On postnatal day 3 (P3), the diencephalon showed much more intense labeling than P1 (Figure 1B), with an increased signal towards the pial borders. At P7, a notable increase in the abundance and distribution of AQP4 transcripts was observed, mainly defined in the glia limitans zones, around the entire cortex, and much more extended toward the dorsal and rostral cortical area (Figure 1C). Intense AQP4 mRNA labeling can be observed in the cerebellum, where the signal concentrates on the external border of the crest, and especially intense labeling accumulates in the deep layers of the crest, those that later develop into the Purkinje cell layer and white matter. Light labeling is observed in the ependymal cells that border the lateral ventricle (inset, Figure 1C′), but very intense labeling occurred above the ventricle, in the corpus callosum (CC, pointed out by the arrow in Figure 1C′).

The ISH image of AQP4 mRNA for a young adult animal (P60) presented in Figure 1D,D′ comes from the Allen Mouse Brain Atlas [19]. There, we can see that the AQP4 transcript is expressed in areas where the presence of the protein is well known for adult mice: the glia limitans, intense expression in ependymal cells in the ventricle, and in the external layers of the cortex (Figure 1D,D′), while the labeling of its mRNA disappears almost completely from the CC, shown by the arrow in Figure 1D′, in contrast to what we observed on P7 (Figure 1C,C′). In the cerebellum, the signal now occurred in the outer layers of the crest, removing the labeling from the most internal white matter zone of the crests in this organ (Figure 1D). That is, in the adult animal, there is an enrichment of the labeling towards cortical areas rich in gray matter, and meanwhile the signal is lost from the highly myelinated areas of the WM.

**Figure 2:**
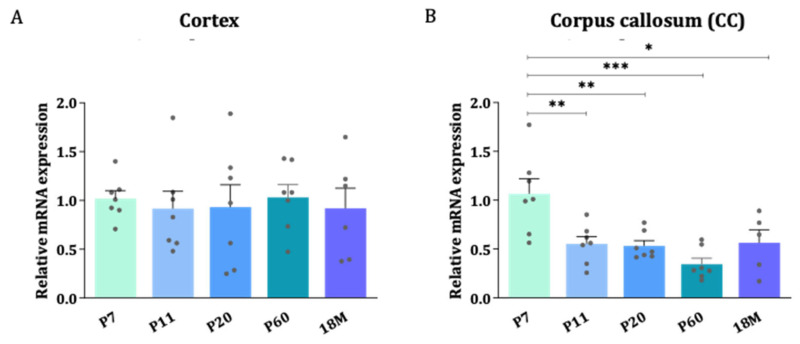
The stability of AQP4 mRNA levels during brain development in the cortex con-trasts with a reduction in gene expression in the corpus callosum. Relative expression changes of AQP4 mRNA measured by RT-qPCR in cortex (**A**) and CC (**B**) across different developmental stages. n = 6–7 animals per group. Mean values are represented with the standard error of the mean (SEM). Statistically significant differences between mean values were assessed by ANOVA test with post hoc TUKEY multiple comparison test (* *p* < 0.05, ** *p* < 0.01, *** *p* < 0.001).

To quantitatively determine the levels of mRNA, the highly sensitive technique RT-qPCR was used. Differences in AQP4 expression between the areas enriched in gray matter (cerebral cortex) and white matter (CC) were evaluated throughout the postnatal development of the CNS (Figure 2A,B). In general, AQP4 mRNA was found to be higher at postnatal ages than in the embryonic phase (see Figure 1 and Supplementary Figure S1), and higher levels after birth were acquired during the first postnatal week (P7). Since the myelinization process, and therefore the clear delimitation of the corpus callosum, is a process that does not start before the first postnatal week [20,21], the first data point in the analysis of the levels of mRNA presented in Figure 2 starts at the age of P7. The data show that from P7 onward, the expression of AQP4 mRNA stabilizes in the cortex (GM) and decreases in the CC (WM) as the mice get older (Figure 2A,B). This reduction observed in the CC was statistically significant between P7 and any other group, but more pronounced compared to P60 levels, where AQP4 mRNA levels fell to 32% of the P7 mean values (*p* < 0.001).

### 2.2. Pattern of Expression of the AQP4 Protein during CNS Development

**Figure 3:**
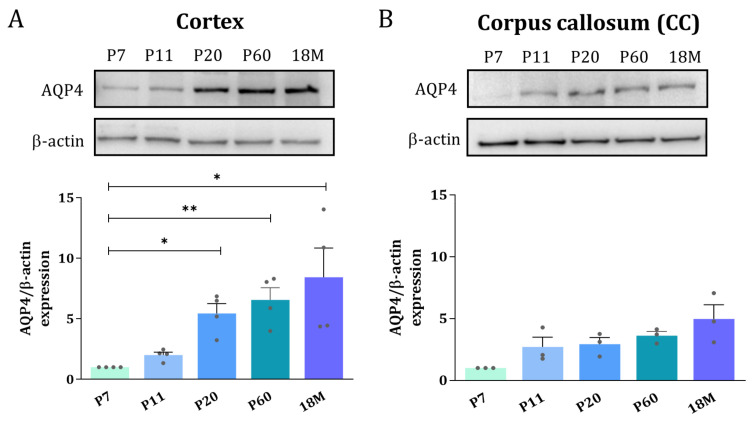
Adult brain undergoes an AQP4 protein increase in the cortex but remains at the same protein levels in corpus callosum. Western blot analysis of AQP4 protein amount at different age groups was performed in the cortex (**A**) and corpus callosum (**B**). Data shown are representative for 3 independent experiments. n = 3–5 animals per group. Mean values are represented with the standard error of the mean (SEM). Statistically significant differences between mean values were assessed by ANOVA test with post hoc TUKEY multiple comparison test (* *p* < 0.05, ** *p* < 0.01).

To explore whether the distribution of the AQP4 protein follows a similar expression inversion pattern between GM and WM as described above for the mRNA, Western blot and immunofluorescence microscopy of AQP4 were performed. Whole protein homogenates from the cortex and dissected areas of CC were analyzed at different ages by Western blot analysis. In the cortex, the levels of the AQP4 protein continuously increase from P7 to 18M, showing a greater than 5-fold increase (* *p* < 0.05) in adult animals (Figure 3A). In the CC, in contrast, no significant differences were detected for the levels of AQP4 protein throughout the wide developmental period studied, although a subtle, similar increase, always larger than 2.5 times, was observed for the ages between P11 and 18M (Figure 3B).

**Figure 4:**
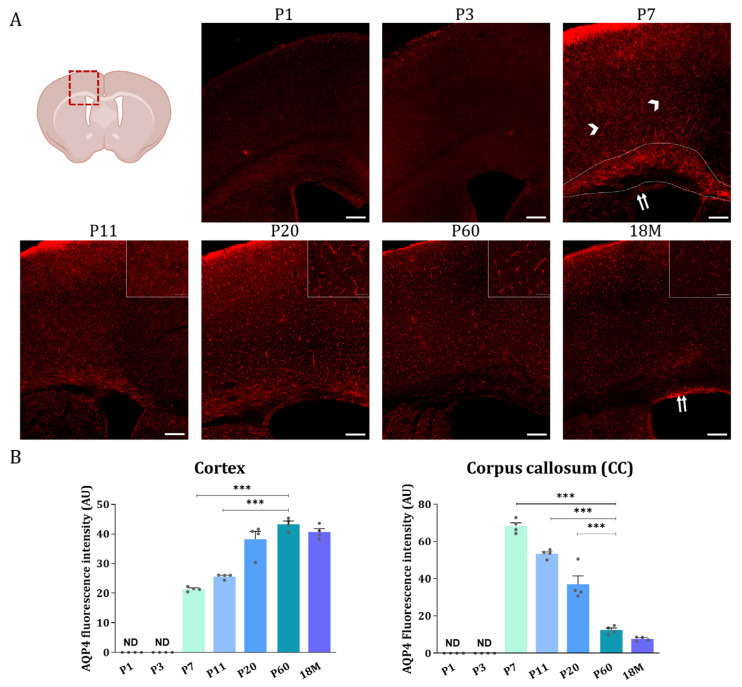
AQP4 immunofluorescence signal shifts from the corpus callosum at early developmental stages to the cortical vasculature in the adult brain. (**A**) Fluorescence Immunohistochemistry showing spatiotemporal AQP4 protein distribution (red) in corpus callosum and cortex from coronal sections at P1, P3, P7, P11, P20, P60 and 18 M. Scale bar = 150 µm. Detailed images of the cortical vasculature were added at P11, P20, P60, and 18M. Inset scale bar = 30 µm. (**B**) Quantification of AQP4 signal was measured by optical density and the intensity levels obtained were compared between different developmental groups in both cortex and corpus callosum regions. Data represent the mean ± SEM from n = 4 analyzed for each age. Statistically significative differences between mean values were assessed by ANOVA test with post hoc TUKEY multiple comparison test. (*** *p* < 0.001). Illustration of the coronal brain section was designed with BioRender (Accessed on 22 November 2022, https://biorender.com/).

Then, AQP4 immunofluorescence analysis was performed on brain sections of animals at different developmental stages (Figure 4). Coronal sections at Bregma 0 are shown in Figure 4A, where the upper wall of the lateral ventricle (LV), the corpus callosum near the top of the ventricle (WM), and the brain cortex (GM) are represented for animals aged 1, 3, 7, 11, 20, and 60 days, and 18 months. As illustrated in Figure 4A, very similar and quite scarce AQP4 labeling was found in all brain regions on postnatal day 1 (P1) and day 3 (P3) (Figure 4, P1 and P3). At these ages, a main difference, not appreciated in this figure but observed at lower magnifications, was that the cortical labeling of AQP4 is no longer restricted to ventral areas and begins to be marked laterally.

At P7, the cortical expression of AQP4 expanded to the lateral and dorsal regions in a similar way, and the signal is still weak but starts to organize around the small vessels (arrow heads, Figure 4, P7). Furthermore, intense P7 labeling of AQP4 was observed along the CC region (area delimited between dashed marks in Figure 4, P7), and expression in ependymal cells bordering the ventricle wall was also detected (double arrow in Figure 4, P7). At P11 (Figure 4, P11), the situation is similar to P7, the labeling of CC is still observed, although with a lower intensity, and the expression pattern in the cortex remains the same. Continuing, at P20 (Figure 4, P20) the vasculature labeling stands out very clearly in the brain cortex, while the signal for AQP4 in the CC remains similar to that detected at P11 (Figure 4, P11). At P60, in a young adult animal, as well as in adult animals at 18M, the expression of AQP4 is no longer detected in the CC (Figure 4, P60 and 18M), however, the cortex and ependyma remain positively stained. Expression of the AQP4 protein in ependymal cells would continue throughout adulthood and even increase with the age of the animal (see the label marked with a doble arrow at 18M in Figure 4, 18M).

Quantification of the AQP4 signal from the immunofluorescence analysis detailed above was by the optical density of representative microphotographs of the different areas of the brain in each stage of development (Figure 4B). The intensity levels of the AQP4 protein show that, in the cortex the expression of AQP4 starts to be detectable at P7, and from there the expression of the protein continuously increases until the adulthood of the animal, a consistent result with the data shown before by western blot (Figure 3A). In contrast, in the corpus callosum the highest levels of the AQP4 protein were detected at P7 (Figure 4B), and from there its levels continuously decreased with the aging of the animal, therefore confirming that the AQP4 protein follows a similar inversion pattern of expression as that described before for its mRNA. i.e., large postnatal expression at P7, in the corpus callosum or white matter tissue, which will diminish or disappear during neurodevelopmental aging, while crescent expression of the protein from P7 through adulthood, in cortical gray matter tissue.

**Figure 5:**
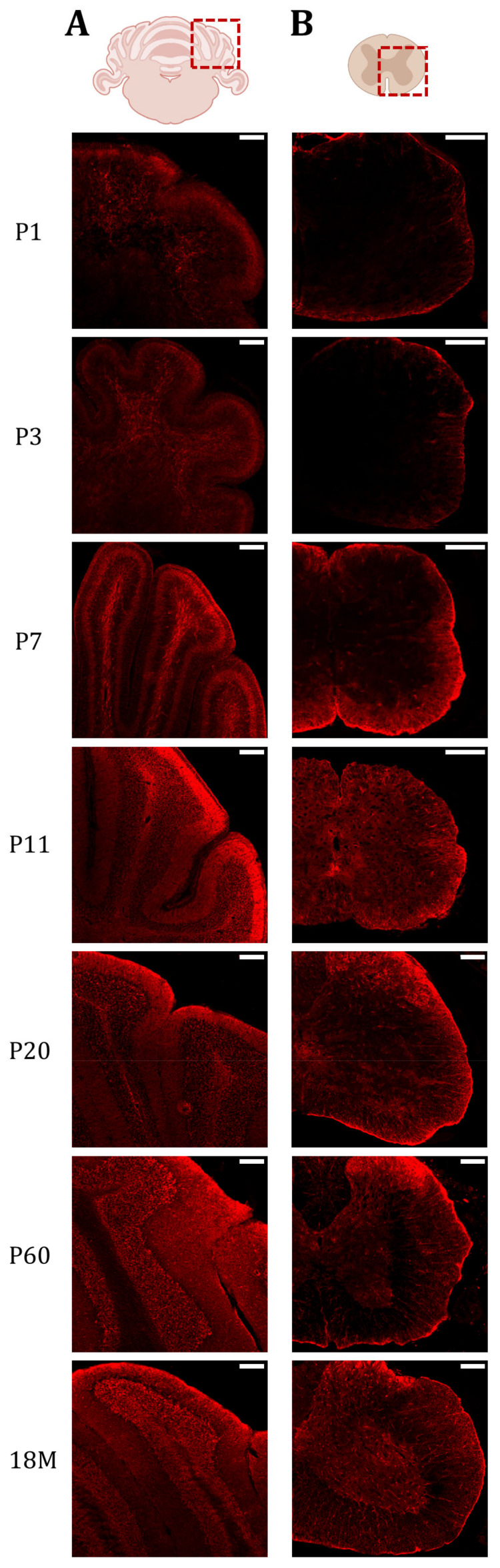
AQP4 protein presence is increased in highly myelinated areas of the cerebellum and spinal cord during postnatal maturation. Fluorescence Immunohistochemistry showing spatiotemporal AQP4 protein distribution (red) in cerebellum (**A**) and lumbar spinal cord (**B**) sections at P1, P3, P7, P11, P20, P60 and 18M. Scale bar = 150 µm. Illustrations of the cerebellum and spinal cord sections were designed with BioRender (Accessed on 22 November 2022, https://biorender.com/).

In the cerebellum and spinal cord, the expression of AQP4 was also analyzed by immunofluorescence (Figure 5). The signal in both organs is relevant for this study because, like the CC, both areas experience a high degree of myelination. In the cerebellum, we were also able to observe the high postnatal expression of AQP4 in the WM of the cerebellum crest at an early age (P7 and P11), and then we could observe how the labeling reduces significantly in this organ in adult animals. At early postnatal ages (P1 and P3) the cerebellum is very poorly developed and the crests are barely recognizable. At these points, the labeling of AQP4 is very weak. At P7, the cerebellum begins to resemble its mature anatomy and AQP4 expression emerges in the deep part of the cerebellar lobules, specifically, in the myelin-enriched internal branches. At P11, this same pattern is present but with a weaker signal intensity, and at P20 the cerebellum shows a lower intensity of fluorescence, remaining at very low levels of expression throughout adult life.

In the lumbar segment of the spinal cord, at P1 (Figure 5, P1), expression is limited to the edges of the spinal cord, and few differences are detected between GM and WM. Something similar was observed at P3 (Figure 5, P3), although the expression of AQP4 in WM begins to be more intense than in the GM zone. At P7 (Figure 5, P7), the difference between both areas becomes very evident, and intense peripheral labeling was observed coincident with the external location of the WM in the spinal cord of animals at this age (P7). When comparing it with P11 (Figure 5, P11), we can see that AQP4 expression in WM decreases significantly, reaching the low levels observed in GM. At P20 (Figure 5, P20), the expression pattern of AQP4 observed in adult life begins to be exhibited, where the highest expression in GM occurs in the dorsal horns, and in WM the expression is strongly reduced (Figure 5, P60 and M18).

**Figure 6:**
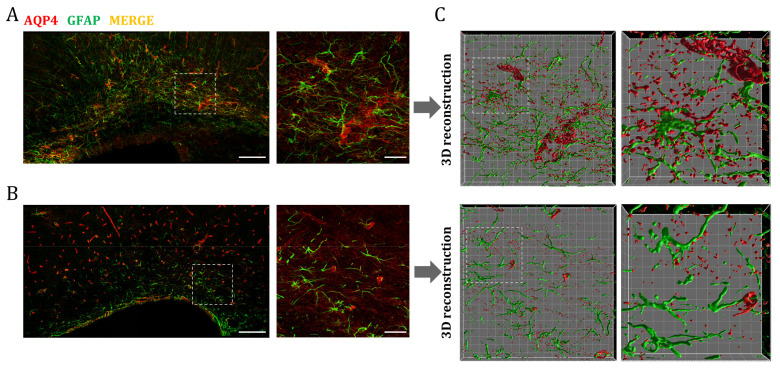
AQP4 overexpression in the postnatal corpus callosum coincides with the emergence of a maturing astrocyte population. Fluorescence immunhistochemistry of GFAP (green) and AQP4 (red) in corpus callosum at P7 (**A**) and P60 (**B**). Higher magnifications show an increased number of astrocytes (GFAP+/AQP4+ cells) at P7 that correlates with postnatal astrocytogenesis. Scale bar = 150 µm; scale bar in the inset = 30 µm. Volumes representing AQP4 and GFAP stained areas in higher magnification were generated by 3D rendering (**C**). Detailed cellular characterizations in each stage indicate differences in glial morphology and AQP4 distribution between premature (p7) and mature fibrous astrocytes (p60). Grid line increment = 5 µm.

Finally, we wanted to identify the cells responsible for the higher expression of AQP4 observed in perinatal WM areas. As already mentioned, astrocytes are equipped with AQP4 in their endfeet processes, so we performed double immunofluorescence staining of AQP4 and GFAP (glial fibrillary acid protein) to determine if the water channel is expressed in these glial cells. As can be seen in Figure 6, a larger population of these GFAP+/AQP4+ cells was visible in the corpus callosum at P7, and exhibited an evident change in the morphology and distribution of AQP4 compared to P60-labeled astrocytes (Figure 6A,B). To better visualize this, 3D surfaces of both the GFAP and AQP4 signals were generated (Figure 6C). This model allowed us to differentiate between a less mature glial phenotype (increased size and more nonspecific distribution of AQP4 in the cell membrane) of the CC at P7, and the adult fibrous astrocyte, with the expression of AQP4 mainly polarized at the endfeet-vessel interface, as is predominantly observed for the CC at P60.

## 3. Discussion

Aquaporin-4 is the most expressed water channel in the central nervous system of mice, as is the case in the rest of the mammalian class. Given its abundant presence in all limitans barriers between plasma, cerebrospinal fluid, and parenchyma, it has been proposed that this protein contributes, associated with its water conductibility, to proper fluid homeostasis in neural tissues. However, the current idea we have today about the distribution and function that AQP4 may have in the CNS comes in general from studies carried out in adult animals. In contrast, the expression and role of this protein during brain development are much less known. Thus, our goal in the present work was to address the expression of AQP4 over time, from a very early postnatal age until adulthood, to uncover the possible participation of this protein in more structural processes, such as those related to astrocyte differentiation necessary for the development of perivascular BBB, the formation of a stable ependymal membrane, and the maturation of the precursors of oligodendrocytes to fully mature cells involved in myelinization.

With ISH, we first analyze the expression of AQP4 mRNA during the first postnatal week. The signal in P1 was located mainly in the brain stem and hypothalamic area (Figure 1A), and even on embryonic day 16, the initial signal in the BS was already observed (Supplementary Figure S1). This pattern was consistent with previous studies, in which an earlier appearance of AQP4 has been observed in the BS and hypothalamus area compared to the cerebellum and cortex [15,21]. Two days later, at P3, the diencephalon has a much more intense labeling than P1, following what others have established, that AQP4 expression begins from the ventral area of the brain and extends to the dorsal area shortly after. Considering that astrocyte differentiation occurs in the form of a ventro-dorsal gradient [14], and since these cells are the ones that express AQP4 the most, their expression pattern will therefore determine the ventro-dorsal pattern observed here for the expression of AQP4. At this stage, the distribution that AQP4 will have in the adult brain begins to be intuitive: glia limitans, ependymal cells, and the subependymal zone; that is, the interface zones between the CNS and spaces containing the CSF. At P7, a much stronger labeling of AQP4 transcripts was observed, and the signal was clearly defined in the glia limitans zones, around the entire cortex, and much more extended towards the dorsal and rostral zones. Furthermore, an important signal of the expression of the AQP4 transcript was detected in the corpus callosum (CC), as shown here (Figure 1C), a location of AQP4 never indicated in adult animals. Therefore, given the availability of ISH images for AQP4 in the Allen Mouse Brain Atlas [19], we decided to observe images of stages similar to those studied in this work. Interestingly, we observed how the marking of AQP4 mRNA in CC was less and less intense at P14 and P28 until it completely disappeared by P60, where it is not marked at all. Furthermore, in this last image (Figure 1D), it can also be seen how AQP4 transcripts are expressed in areas where their presence has been indicated in adults: glia limitans, ependymal cells, and around vessels. That is, in the adult brain of mice there is an enrichment of the AQP4 labeling towards the cortical areas, and it is lost in the highly myelinated area of the CC. Quantification, by RT-qPCR, of AQP4 levels in the cortex and in the CC (Figure 2A,B), also highlighted that the highest expression of AQP4 in mice brains appears to occur during the perinatal period, reaching a peak at P7, and reducing its expression towards adult life. This reduction is especially important in white matter (CC), where expression levels drop by more than 80% (*p* < 0.05, Figure 2B) in the adult brain. On the contrary, the levels of expression in the cortex remain constant throughout development, after reaching a peak at P7. In adult mice, the expression of AQP4 is different between protoplasmic astrocytes (PA) and fibrous astrocytes (FA) [17,18,22]. Basically, in the GM, AQP4 is expressed in PA, which has long branched processes and is marked with S100β. In WM, AQP4 is expressed in FA, which have short branched processes and a greater expression of GFAP.

During postnatal development, the radial glia detach from the subventricular zone to bring their soma to the pial surface, and at this point differentiation into PA or FA takes place. From P7 to P21, the astrocytes go through a transcriptional program that ensures their functional maturity, which is also associated with morphological changes such as a smaller size, becoming less branched, and presenting long extensions from the soma with a filopodia structure. At P21, these filopodia structures disappear and their characteristic fine extensions appear [18]. As we see in our results (Figure 2), P7 is the point where the expression of AQP4 mRNA is at its maximum in the CC, therefore, it is possible that AQP4 is involved in this fibrous astrocyte differentiation process. To support this, we showed, by immunofluorescence analysis of the GFAP and AQP4 signals (Figure 6), how this overexpression is associated with a less mature astrocyte subset expressing AQP4 in an unpolarized way. Some authors have proposed that AQP4 expression may be the first marker for the intermediate gliogenic progenitor initiating the development of the astrocytic program [23]. Our study would support this idea by confirming the increased expression of AQP4 in the CC at the stage where developmental astrocytogenesis reaches its highest rates.

In addition to this, facilitation and maintenance of myelination in the CNS, carried out by oligodendrocytes in the axons of neurons, are also included among the many functions performed by these astrocytes. FAs of the WM are specialized in this process and participate in it by removing ions and neurotransmitters from the extracellular space, and secreting pro-myelinating factors. During CNS development, the first mature astrocytes are detected at stage E16; this coincides with the beginning of AQP4 expression, as shown in this work (Supplementary Figure S1) [14]. Myelination in rodents is scarce before birth and is accelerated during the first postnatal stages [17]. Again, coincident with this, our results evidenced a peak of AQP4 expression, at the age of P7, in the WM (CC, cerebellum crest and in the spinal cord), compared to the cortex GM, allowing us to propose that AQP4 expression must be associated with mature FA that would stimulate oligodendrocytes to myelinate axons in these WM zones. Regarding this hypothesis, a population of AQP4 positive glial stem cells has recently been identified in the fibers of the white matter tract of developing human brains [24]. The emergence of this population was dated at the beginning of the third trimester (25 PCW), just before myelination takes place in human brains [25], and according to the authors, these cells would provide a scaffold for glioblasts that will develop and participate in axon maturation and myelination [23]. Interestingly, this population was shown to be altered in congenital hydrocephalus, a disease associated with corpus callosum abnormalities [24].

When the astrocytes are dysfunctional, it affects the oligodendrocytes and therefore the myelination of the tissue. Thus, in KO mice for GFAP, one of the proteins that gives integrity to the cellular structure of astrocytes, the animals present many defects in the structure and composition of the WM [26]. Then, it seems appropriate to propose that the increase in AQP4 expression in WM astrocytes at P7 should be related to the CNS myelination process that occurs at this postnatal age in the mouse. The cellular distribution of the AQP4 protein in the brain is dynamic and, under some conditions, can even be independent of mRNA expression levels [27,28,29], as seen here in the CC. Alternative splicing has been described that leads to 4–6 distinct transcripts for AQP4 in the brain of mice [28], increasing the options for protein expression and regulation.

Furthermore, novel ways of regulating the AQP4 protein in brain tissues, such as parenchyma, that exclusively depend on post-translational mechanisms of recycling to the membrane via the KIDINS220-SNX27-retromer-AQP4, have recently been described [29]. Therefore, in the present work, we also looked at the postnatal distribution of the AQP4 protein directly, to confirm the results previously described based on the analysis of AQP4 mRNA. Our results (Figure 3 and Figure 4) confirm that, similarly to what is seen for mRNA, the AQP4 protein exhibits a peak of expression in the CC at the age of P7, the levels remain high up to P11, and then start to decrease until complete disappearance from the CC in the adult animal at P60 (Figure 3). Meanwhile, protein levels in the cortex remain low until P20, after which time the signal starts to organize around small blood vessels and remains with this perivascular pattern throughout adulthood. This redistribution of the protein, which was initially expressed in WM (CC, internal area of the cerebellum crests and external zone in the spinal cord) during the first week of the postnatal period (P7-P11 in Figure 3 and Figure 4), switching off and then appearing in the GM areas (brain cortex, external area of the cerebellum crest, and dorsal horns of the spinal cord) of the adult animal (P60 and 18M in Figure 3, Figure 4 and Figure 5), suggests the participation of AQP4 in important, but different, processes throughout the life of the animal. Among these processes in the postnatal stage, the presence of AQP4 has been associated with buffering the amount of water in the brain as the extracellular space is reduced [13], helping the BBB mature, or facilitating osmoregulation of specific regions [15]; but in the young adult stage (P7 to P20), we propose that AQP4 expression is more related to the myelination process of the CC and cerebellum-type tissues. It should be noted that the appearance of AQP4 in areas of high myelination coincides with the moment of greatest oligodendrogenesis, that is, the production of the cells responsible for myelination of the CNS. Therefore, the association of AQP4 with this process seems more than plausible. More studies addressing the role of AQP4 in the myelination process along the maturation of the CNS are needed to improve our understanding of the novel roles of AQP4 in the neural development in mammals.

## 4. Materials and Methods

### 4.1. Animals

C57BL/6 male and female mice were housed in a regulated temperature environment (22 ± 1 °C) in a 12 h light/dark cycle, with ad libitum access to food and water. The general health status of the experimental mice was analyzed by daily observation. Mice were sacrificed under deep anesthesia induced by a combination of 100 mg/kg of ketamine (Pfizer, New York, NY, USA) and 10 mg/kg of xylazine (Bayer, Leverkusen, Germany). All experiments were carried out in accordance with the European Directive 2010/63/EU and the Spanish RD/53/2013 on the protection of animals used for scientific purposes. Animal procedures were approved by the Animal Research Committee of the Virgen del Rocio University Hospital (26/01/2017/017; University of Seville).

### 4.2. RNAscope^®^ In Situ Hybridization (ISH)

In situ hybridization with the RNAscope^®^ technique was performed according to the manufacturer’s instructions (Advanced Cell Diagnostics (ACD), Newark, CA, USA) in paraffin-embedded sections of aortic rings previously fixed with 4% PFA (paraformaldehyde). Five micrometer thick sections were deparaffinized and treated with H_2_O_2_ followed by antigen retrieval and protease treatment, according to the kit instructions (RNAscope H_2_O_2_ & Protease Plus reagents ACD #322330). Mice from embryonic day 16 (E16) and postnatal days 1, 3, and 7 (P1, P3, P7) were anesthetized and perfused intracardially with phosphate buffered saline (PBS, Sigma, St. Louis, MO, USA) and 4% paraformaldehyde (PFA, Sigma) in PBS, with a volume between 10 and 50 mL depending on the stage of development and the size of the animal. Subsequently, the brains were immediately removed and fixed with 4% PFA in PBS for 2 h at 4 °C, cryoprotected in 30% sucrose in PBS at 4 °C for 24 h, and embedded in the optimal cutting temperature (OCT) compound (Sakura Finetek, Torrance, CA, USA) prior to storage at −80 °C. 20 µm sagittal brain slices were obtained with a cryostat (Leica, Wetzlar, Germany) and stored at −80 °C until use. In situ hybridization by the RNAscope^®^ technique was performed according to the manufacturer’s instructions (Advanced Cell Diagnostics (ACD)) for fixed frozen tissue sections. Antigen retrieval and protease treatment were carried out according to the kit instructions (RNAscope H_2_O_2_ & Protease Plus ACD #322330). AQP4 probes (ACD #417161) were hybridized for 2 h followed by six amplification steps, using a HybEZ oven (ACD). The signal was detected with the Brown RNAscope 2.5 HD detection kit (ACD #322310), with an incubation time of 15 min. The slices were mounted with Fluoromount-G mounting medium (Invitrogen, Waltham, MA, USA) and analyzed in wide-field microscopy with a Leica DMi8 inverted microscope with THUNDER computational clearing software.

### 4.3. RNA Extraction and Quantitative Reverse Transcription PCR Analysis

The cortex and corpus callosum of animals from P7 to 18 months were microdissected in ice cold diethyl pyrocarbonate (DEPC)-PBS under a stereoscopic binocular microscope (Olympus SZX16, Tokyo, Japon) from fresh brain coronal sections (thickness 1 mm). For the cortex and corpus callosum of P7, P11, and P20 animals, total RNA was isolated using the RNeasy Micro Kit (Qiagen, Hilden, Germany). For highly myelinated corpus callosum of P60 and 18-month animals, total RNA was isolated using first Trizol reagent (Invitrogen) to separate the aqueous phase, which was then transferred to a RNeasy MinElute spin column of the RNeasy Micro Kit and continued according to the manufacturer’s instructions. cDNA synthesis was performed using the QuantiTect reverse transcription kit (Qiagen, Hilden, Germany), and AQP4 relative mRNA expression levels were measured using quantitative real-time polymerase chain reaction (RT-qPCR) with the ViiA 7 Real-Time PCR System (Thermo Fisher, Waltham, MA, USA). AQP4 mRNA expression levels were normalized using 18S ribosomal mRNA to correct for variations in the amounts of RNA input, and all samples were analyzed in triplicate. The Primer Express software v2.0 (Applied Biosystems, Waltham, MA, USA) was used to design primers AQP4-F (3′-CCGTCTTCTACATCATTGCACAGT-5′), AQP4-R (3′-GCGGTGAGGTTTCCATGAA-5′),18S-F (3′-AACGAGACTCTGGCATGCTAA-5′) and 18S-R (3′-GCCACTTGTCCCTCTAAGAAGT-5′). The quantity and purity of RNA and cDNA were assessed with a NanoDrop ND-1000 UV–vis spectrophotometer (Thermo Fisher).

### 4.4. Immunohistochemistry (IHC)

The brains of the animals at E16, P1, P3, P7, P11, P20, P60, and 18 months were fixed and obtained as previously described, cryoprotected in 30% sucrose in PBS, and included in OCT (Tissue-Tek). The spinal cords were additionally decalcified with 10% EDTA in PBS before the sucrose cryoprotection step. Coronal sections of 30 µm were cut on a cryostat (Leica, Wetzlar, Germany). The immunofluorescence of fibrillary acid protein (GFAP) and AQP4 was performed as previously described [30,31], using monoclonal anti-GFAP (1:300; Sigma) and polyclonal AQP4 (1:100; α-diagnostic, San Antonio TX, USA). Anti-mouse IgG conjugated with Alexa Fluor488 (1:400; Invitrogen) and anti-rabbit IgG conjugated with Cy3 (1:200; Jackson-Immunoresearch, Cambridgeshire, UK) were used as secondary antibodies for GFAP and AQP4, respectively. The tissue slices were mounted with Fluoromount-G mounting medium. Simple fluorescent images of AQP4 were visualized using a Leica DMi8 inverted microscope, and GFAP colocalization images were acquired using a confocal microscope (Leica Stellaris 8) in a 0.5-um Z-stack series.

### 4.5. Imaging Quantification and 3D Reconstruction

To quantify the abundance of AQP4 protein in the coronal sections, the optical density of the AQP4 + staining was measured using rhe NIH Image software (ImageJ, version 1.53t). For this study, three slices per mouse were included. These slices were representative of the cerebral region comprised between +0.62 and −0.10 mm rostro-caudal relative to the Bregma point in the Franklin and Paxinos mouse brain stereotaxic atlas. After defining the properties for a specific ROI, the area of the explored regions (corpus callosum and cortex) was randomly chosen and quantified. To perform the 3D reconstruction analysis of the AQP4/GFAP immunofluorescence micrographs, the software Imaris (Bitplane, version 6.0) was used.

### 4.6. Protein Extraction, Quantification, and Western Blot

After collection, tissues were homogenized in lysis buffer containing 50 mM HEPES, pH 7.3, 250 mM NaCl, 5 mM EDTA, 0.2% (*v*/*v*) NP-40, 5 mM dithiothreitol and 1% (*v*/*v*) of a cocktail of protease inhibitors (Sigma). Tissue disruption was achieved by briefly using a dounce homogeneizer, and tissues were left at 4 °C for 15 min. The samples were then centrifuged at 16,000× *g* for 20 min at 4 °C and the supernatants containing proteins were isolated. The Bradford method was used to determine protein concentration, and the samples were kept at −20 °C until Western Blot analysis. 15 µg of protein lysates were boiled for 5 min at 95 °C, and then loaded onto PRECAST gels. After electrophoresis, the proteins were transferred to PVDF membranes (Hybond-P, Amersham Biosciences, Amersham, UK) using the wet chamber method. The membranes were probed with polyclonal anti-AQP4 (1:1000; Alomone, Jerusalem, Israel) or monoclonal anti-β actin (1:10,000; Abcam, Cambridge, UK) antibodies. Immunoreactive bands were developed with ECL (Bio-Rad, Hercules CA, USA) and visualized using a digital imaging system (I.Q. LAS 4000 MINI GOLD, GE HealthcareQ9, Chicago, IL, USA).

### 4.7. Statistical Analysis

Data are presented as mean ± standard error of the mean (SEM), and the statistical test performed is specified in each figure legend. For all the analyses performed, the data were tested for normality (Kolmogorov–Smirnov test) and equal variance. When these properties were confirmed, variance analysis was performed with Tukey’s HSD post hoc analysis for multiple group comparisons or Student’s t test (for two-group comparisons); otherwise, the nonparametric Kruskal–Wallis H test (for multiple comparisons) or Mann–Whitney U test (for two-group comparisons) was used. All statistical analyses were performed with the Graphpad Prism, version 8.4.2., San Diego, CA, USA.

## 5. Conclusions

We showed that AQP4 mRNA expression is increased in postnatal development compared to adult life in mice, particularly in WM areas where it reaches its maximum on postnatal day 7. These higher expression levels were correlated with protein abundance at this point, prior to the onset of developmental myelinization in the tissue. AQP4’s presence in white matter areas extended from P7 to P20, a period that overlaps with active oligodendrogenesis. It is suggested that AQP4 could be located in fibrous astrocytes maturing in WM that contribute to the precursor cells necessary for myelin formation. AQP4 expression in GM areas increases throughout development, located at the feet of protoplasmic astrocytes surrounding the vasculature. Its presence in this location is associated with the need for neuronal somas for greater vascularization to obtain more oxygen and nutrients.

## Data Availability

Not applicable.

## Supplementary Materials

The following supporting information can be downloaded at: https://www.mdpi.com/article/10.3390/ijms24033048/s1.
