# Supplementary material for: Aquaporin-4 Expression Switches from White to Gray Matter Regions during Postnatal Development of the Central Nervous System
Source: Int J Mol Sci. 2023 Feb 3;24(3):3048. doi: 10.3390/ijms24033048 (PMC9917791; doi:10.3390/ijms24033048)

Supplementary Figure S1. Identification of AQP4 mRNA transcripts by in situ hybridization in cerebral sagittal section from E16.0 embryos. Scale bar = 1 mm**.**

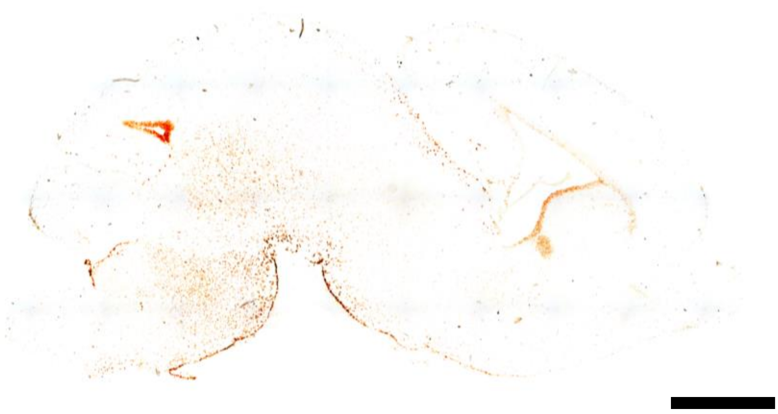

Supplement: Supplementary file 1 [file ijms-24-03048-s001.zip › ijms-2173781-supplementary.pdf]
